# 

**Published:** 1858-04

**Authors:** 


### RECEIPTS ON SUBSCRIPTIONS,

UP TO APRIL 14TH, 1858.

### ILLINOIS.

| | | |
|---|---|---|
| Dr. S. H. Culver, | VOL. 5 & 6, | $4 00 |
| " Z H. Madden, | " 6, | 2 00 |
| " J. W. York, | " 6, | 2 00 |
| " Hosea Davis, | " 6, | 2 00 |
| " R. S. Hitt, | " 6, | 2 00 |
| " G. A. Byrnes, | " 2 & 3, | 5 00 |
| " Benj. Durham, | " 1, | 2 00 |

### IOWA.

| | | |
|---|---|---|
| Prof. J. H. Rauch, | VOL. 1, | $2 00 |

### INDIANA.

| | | |
|---|---|---|
| Dr. O. H. P. Evans, | VOLS. 3,4, 5,6 & 1, | $10 00 |
| " S. C. Gray, | " 6, | 2 00 |
| " G. F. Somers, | " 1, | 2 00 |
| " J. H. Buell, | " 6, | 1 00 |
| " W. Vannuys, | " 5, | 2 00 |

### MICHIGAN.

| | | |
|---|---|---|
| Dr. G. P. Alger, | VOL. 3, | $2 00 |
| " C. S. Tucker, | " 1, | 2 00 |

